# Dry Electrode-Based Body Fat Estimation System with Anthropometric Data for Use in a Wearable Device

**DOI:** 10.3390/s19092177

**Published:** 2019-05-10

**Authors:** Seung-Chul Shin, Jinkyu Lee, Soyeon Choe, Hyuk In Yang, Jihee Min, Ki-Yong Ahn, Justin Y. Jeon, Hong-Goo Kang

**Affiliations:** 1 The Department of Electrical and Electronic Engineering, Yonsei University, Shinchon-dong, Seodaemun-gu, Seoul 03722, Korea; sc1225.shin@yonsei.ac.kr (S.-C.S.); shuya@dsp.yonsei.ac.kr (J.L.); schoe@dsp.yonsei.ac.kr (S.C.); 2 The Department of Sport Industry Studies, Yonsei University, Shinchon-dong, Seodaemun-gu, Seoul 03722, Korea; hyukin.yang@yonsei.ac.kr (H.I.Y.); jihee8700@yonsei.ac.kr (J.M.); jjeon@yonsei.ac.kr (J.Y.J.); 3 The Faculty of Kinesiology, Sport, and Recreation, University of Alberta, 1-115 University Hall, 116 St. and 85 Ave., Edmonton, AB T6G 2R3, Canada; kiyong1@ualberta.ca

**Keywords:** bioelectrical impedance analysis, deep learning, percent body fat, upper-body measurement, settling value estimation

## Abstract

The bioelectrical impedance analysis (BIA) method is widely used to predict percent body fat (PBF). However, it requires four to eight electrodes, and it takes a few minutes to accurately obtain the measurement results. In this study, we propose a faster and more accurate method that utilizes a small dry electrode-based wearable device, which predicts whole-body impedance using only upper-body impedance values. Such a small electrode-based device typically needs a long measurement time due to increased parasitic resistance, and its accuracy varies by measurement posture. To minimize these variations, we designed a sensing system that only utilizes contact with the wrist and index fingers. The measurement time was also reduced to five seconds by an effective parameter calibration network. Finally, we implemented a deep neural network-based algorithm to predict the PBF value by the measurement of the upper-body impedance and lower-body anthropometric data as auxiliary input features. The experiments were performed with 163 amateur athletes who exercised regularly. The performance of the proposed system was compared with those of two commercial systems that were designed to measure body composition using either a whole-body or upper-body impedance value. The results showed that the correlation coefficient ($r^{2}$) value was improved by about 9%, and the standard error of estimate (SEE) was reduced by 28%.

## 1. Introduction

Many healthcare systems are designed to continuously monitor users’ health condition or to pre-diagnose disease. Thanks to the development of sensor technology and communication technology, the healthcare market has been growing rapidly [1]. In this application, effectively manipulating a large amount of data is very important; thus, the role of machine learning-based technologies has become crucial. Among many types of biometric information, body composition is one of the most fundamental and essential parameters for checking each person’s health condition. For example, excessive fat in the human body is closely related to diseases such as obesity, diabetes, and even cancer [2,3,4].

Dual-energy X-ray absorptiometry (DEXA) has been widely used to analyze body composition because of its high measurement accuracy [5,6,7]. However, it is not suitable for everyday use because the size is too large and the device must be operated by a specialist. To overcome these problems, bioelectrical impedance analysis (BIA) techniques have been widely used. These techniques utilize variations of electric conductivity, which varies by the composition of human substance and impinged frequencies [8,9]. Specifically, an impedance value is measured using an electrode attached to the human body. Then, the percent body fat (PBF) is predicted by a pre-trained mapping rule between impedance and PBF. In general, a whole-body impedance-based technique [10] shows higher accuracy than an upper-body impedance-based one [11]; however, it is not suitable for implementing a portable-type device because it requires many electrodes [12,13]. In addition, the size of conventional upper-body impedance-based methods is too large for use with a wearable device.

In this study, we developed a hardware device and a software algorithm to easily and accurately estimate PBF using upper-body impedance and auxiliary anthropometric features. To make the device convenient for wearable applications, we designed an 8×8 mm^2^ stainless steel electrode. The small size of this electrode has a narrow contact area with the skin; thus, it takes a very long time to obtain a stabilized impedance value. To address this problem, we propose a model-driven algorithm with a settling time of only five seconds. To improve the comfort of the measurement posture, we used the wrist of one hand and the index finger of the opposite hand as skin contact points [14]. The reason for choosing the index finger method is that the area where the left and right hands overlap with each other in the measurement process is smaller than in other conventional wearable devices [15]. In this method, the measured impedance value is highly influenced by the impedance value of the index finger; thus, it is not suitable to use the conventional cylinder-based model [8,16]. The cylinder-based model, whose target is determined by the value of $Height^{2}/R$, where R denotes impedance, only utilizes information from the trunk, arms, and legs, but not that of the hands and feet. In this paper, we updated the $Height^{2}/R$ prediction algorithm to reduce the effect caused by the index finger impedance. In addition, we estimated PBF by finding a mapping rule between the calibrated impedance value and the true PBF.It should be noted that the information obtainable from the upper-body impedance measurement system is insufficient to predict the whole-body PBF. To address this problem, waist and hip circumferences were used as additional input features. Typically, the mapping rule is implemented by conventional linear regression techniques [8,16], but we introduced a deep learning-based regression framework to achieve higher accuracy.

We first collected data from 163 university athletes with various expertise and volunteers who exercised more than four times a week. Then, we compared the performance of the proposed system with those of two commercial systems utilizing either whole-body [12] or upper-body impedance value [11]. The experiments confirmed that the proposed system showed much higher PBF estimation accuracy than conventional methods, while its measurement time was only five seconds. The results confirmed that small electrodes could be used to accurately measure PBF, thereby demonstrating the feasibility of designing various types of wearable devices.

## 2. Related Works

### 2.1. Various Types of Body Composition Measurement Methods

Obesity can be diagnosed instantaneously by body mass index (BMI) or waist-to-hip ratio [4,17,18]. These methods are easy to use, but their accuracy is low, mainly because their assessment is based purely on height, weight, waist, and hip circumferences, and not body composition [19]. Body composition can be accurately measured in a variety of ways [7,20]. In the past, underwater weighing and air-displacement plethysmography were used to determine PBF. Because these methods utilize the density of the human body in a well-controlled environment, they are inconvenient and have space constraints. A skin-folding method that measures the thickness of subcutaneous fat requires a simple tool, but expert guidance is needed for precise use [21]. The current gold standard is an image analysis technique using DEXA [5], which measures bone, fat, and lean body mass via X-rays. However, it is only used in hospitals because DEXA requires X-ray telegraphy equipment.

### 2.2. Body Composition Measurement Using Bioelectrical Impedance Analysis (BIA)

**Figure 1:**
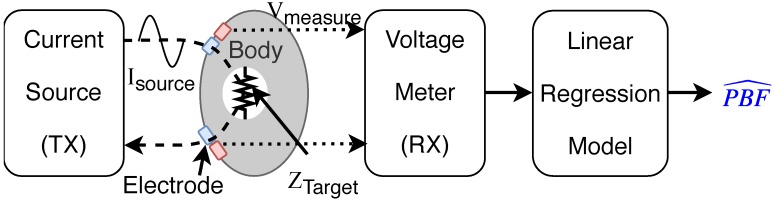
Bioelectrical impedance analysis (BIA) measurement principle. The electrode is in contact with the skin, and the frequency of the current source differs by the purpose of the measurement. To obtain a stabilized constant impedance value, the measurement time needs to be set sufficiently long in general.

Another measurement method is BIA, which uses the difference of electric characteristics in the body cell structure depending on the frequency of impinged current. In the method, a current having a specific frequency is passed through the human body, and the impedance value is calculated by measuring the voltage as shown in Figure 1 [22]. In general, a frequency range between 1 kHz and 1 MHz is used to provide a detailed assessment of body composition [23]. Since the components that pass through the body differ depending on the frequency range, it is possible to analyze multiple factors such as total body water (TBW), fat-free mass (FFM), and fat mass (FM) [16,24,25,26]. To estimate PBF, various regression models that utilize impedance values, height, age, gender, ethnicity, and weight parameters have been proposed [27,28]. In a simple cylinder-shaped model [8,16], the $H^{2}/R_{50}$ value is used for predicting PBF, where *H* is the height and $R_{50}$ is the impedance value measured by the impinging frequency of 50 kHz. Various types of regression models have been proposed, which only differ in their experimental setups and subjects’ characteristics [29,30,31,32,33,34,35,36]. Previous studies have reported that they could obtain a standard error of estimate (SEE) ranging from 1.8 to 8.8, and a correlation coefficient, i.e., $r^{2}$, ranging from 0.71 to 0.97 [29,30,31,32,33,34,35,36]. It is well known that the performance varies by differences in the parameters used rather than the relative superiority of any method over others. Nonetheless, compared to the DEXA and densitometry methods, the BIA measurement method is a practical alternative.

### 2.3. Effect of Measurement Position on BIA Method

**Figure 2:**
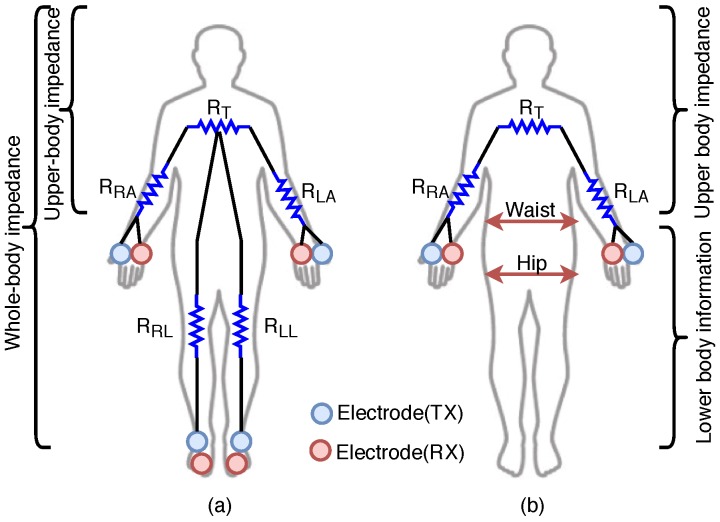
Measurement information and electrode configuration. Each measurement point has two electrodes. One is TX for current injection, and the other is RX for voltage sensing. (**a**) Conventional measurement parameters: Five impedance measurements using eight electrodes; (**b**) proposed measurement parameters: Five measurements using four electrodes.

**Figure 3:**
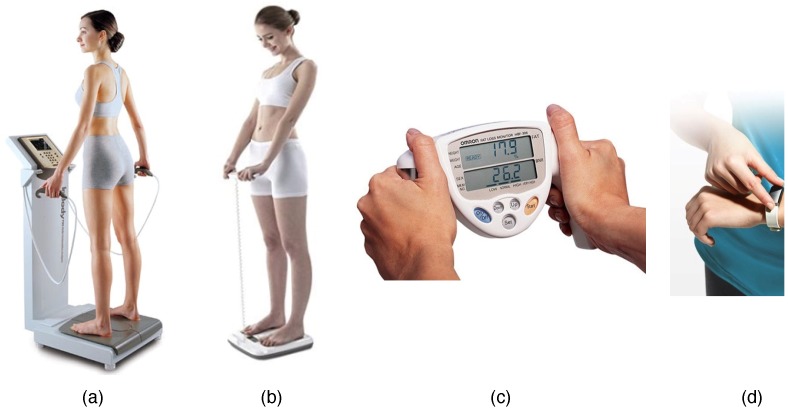
Examples of commercial percent body fat (PBF) measurement devices using the BIA method. The devices can be classified into either whole-body or upper-body type depending on the measurement methods, and either hand-held or wearable type. (**a**) Inbody-720: Inbody Co., Ltd. (Seoul, Korea), Whole-body impedance measurement, 8-electrode [12]; (**b**) Scale type: Tanita Co., Ltd. (Tokyo, Japan), Whole-body impedance measurement, 8-electrode [13]; (**c**) HBF-306: OMRON Co., Ltd. (Kyoto, Japan), Upper-body impedance measurement, 4-electrode [11]; (**d**) Inbody band: Inbody Co., Ltd., Upper-body impedance, 4-electrode [15].

Body-impedance measuring devices can be classified into two types: Whole-body or upper-body, as shown in Figure 2a. A whole-body measurement device predicts body composition after measuring impedance in the arms ($R_{RA},R_{LA}$), legs ($R_{RL},R_{LL}$), and torso ($R_{T}$), as depicted in Figure 2a. Typically, this method uses eight electrodes, two on each hand and two on each foot as shown in Figure 3a,b [12,13]. The measurement takes approximately three minutes [12]. Because the measurement posture used as Figure 3a is more stable, various body configurations can be measured using multifrequency techniques, which include fat free mass (FFM), total body water (TBW), mineral, and abdominal fat. However, since the device used for measuring body impedance requires a footplate with four electrodes, the size of the device must be at least 300×300 mm^2^, which makes it difficult to carry [13]. To increase portability and the ease of taking measurements, an upper-body device was developed as shown in Figure 3c. This technique measures the impedance value between the left and right hands [11]. Based on this, body composition is calculated with the same parameters used in the whole-body measurement method. The size of the device used to measure upper-body impedance is 197×49×128 mm^3^, and it includes four electrodes, each of which is 35×40 mm^2^. Because this device includes four large-sized electrodes, the time required for a measurement is only three seconds; however, it is also difficult to implement it in a wearable form. Finally, Figure 3d shows a wearable type of device to be designed for a portable device. It uses one hand’s wrist and the other hand’s thumb and index finger.

### 2.4. Electrode Separation Effect between TX and RX

**Figure 4:**
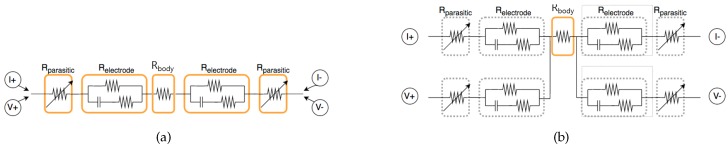
Modeling measurement parameters by the configuration of TX and RX. (**a**) TX and RX sharing case; (**b**) TX and RX separation case.

As mentioned in Section 2.3, two electrodes are used at each measurement site to separate the electrodes of TX (current injection) and RX (voltage sensing). Based on the system that measures BIA, the device and the human body loop can be modeled as shown in Figure 4. The first one is the $R_{parasitic}$ component that models the impedance between the electrode and skin, the value of which is mostly influenced by the state movement of skin. Next one is the $R_{electrode}$ generated in the electrode itself, which is determined by the material and the composition of the electrode. Finally, an impedance of $R_{body}$ value is measured. Note that the variation of $R_{parasitic}$ caused by motion can be measured in the TX and RX shared system as shown is Figure 4a because it uses the same current and voltage. However, in the case of the 4-electrode system as shown in Figure 4b, it is possible to eliminate the influence of the variation of $R_{parasitic}$ because the current and voltage paths are operated separately. For this reason, many commercial products use 4 electrodes. In this study, the electrode was also constructed in a form of separating TX and RX.

The novelty of the proposed system is that it predicts PBF quickly and accurately using only upper-body impedance, measured by a small electrode. To implement this system, we developed an algorithm that minimizes impedance-measurement time and a modeling technique that accurately predicts PBF by using supplementary waist and hip circumferences information as shown in Figure 2b.

## 3. System Design and Data Acquisition Method

**Figure 5:**
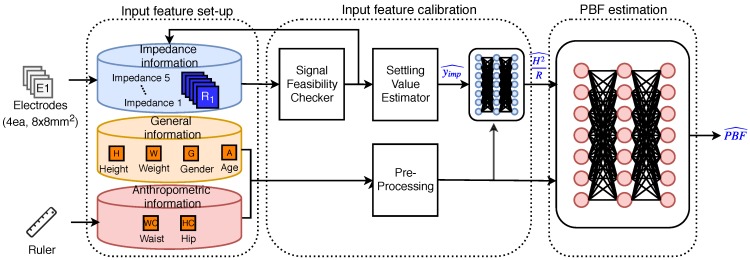
Block diagram of overall system structure. The input features consist of body information and impedance values measured for five seconds. The measured impedance values are calibrated to improve accuracy. Finally, a deep neural network (DNN) is used to predict PBF accurately.

Figure 5 shows the overall structure of the proposed system, which consists of an input feature–setup module, an input feature–calibration module, and a deep learning-based PBF-prediction module. In the input feature–setup module, body impedance is measured using the developed small-sized sensing device explained in the previous section. The input feature–calibration module is needed to minimize measurement time and error. Furthermore, we included waist and hip circumference values into the input features to improve accuracy. In addition, we adopted a deep learning-based model to faithfully represent the relationship between input features and output parameter values.

### 3.1. Hardware Design and Measurement Posture

**Figure 6:**
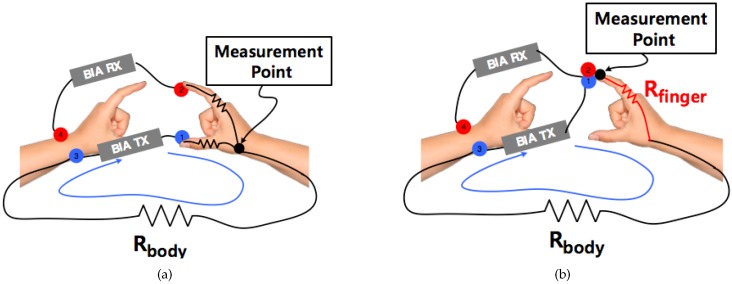
The upper-body impedance-measurement method using a wearable device. This method uses four electrodes: Two as the current source (TX) and two to measure voltage (RX). One RX and one TX electrode are placed at each measurement site. (**a**) wrist + two-finger type; (**b**) wrist + one-finger type.

In recent years, small wearable-type devices with four electrodes have been developed to comfortably measure body composition [15,37,38]. To realize an upper-body feedback loop, two electrodes play a role in making the current flow from the inside of the device to the wrist, and the other two electrodes from the outside of the device. These devices are categorized into three types depending on the contact point of the electrodes, such as palm grip holding posture, one-finger method, and two-finger method. The palm grip posture is used for large-sized electrode-based devices because the devices need to be stably contacted. Because the electrode size of the wearable device is small, it is generally implemented using a finger contact type. The two-finger device depicted in Figure 6a takes measurements from the thumb and the index finger [15]. This method returns the same impedance value as the conventional palm grip holding posture because it measures the intersection of the RX and TX as shown in Figure 6a. Therefore, we still used the same modeling technique as the one used in previous studies. To ensure an accurate measurement, no direct contact should be made between the left and right hands. However, when placing the right thumb and index finger on the left wrist, it is difficult for the left hand to avoid touching the right hand.

**Figure 7:**
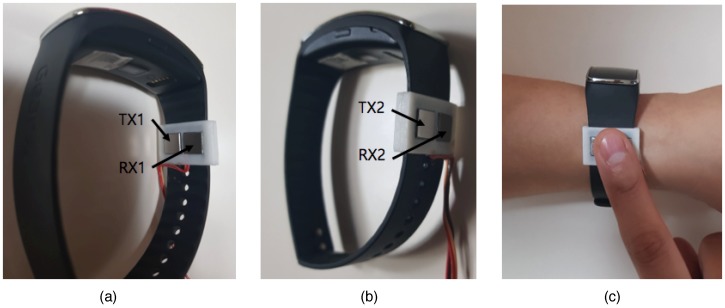
An example hardware design and measurement posture (**a**) electrode configuration installed at the inside of the band consisting of TX1 and RX1 electrodes; (**b**) electrode configuration installed at the outside of the band consisting of TX2 and RX2 electrodes; (**c**) measurement posture using the device. Electrodes are connected to the external board via a wire which consists of a TX and RX circuit.

To overcome this problem, we only used one index finger as a contact point, as shown in Figure 6b [14]. This is a more effective way to prevent direct contact between the left and right hands. Figure 6a,b is similar in terms of measurement posture, but the characteristics of the measured data differ because their body-path elements are different. Because the measured value includes the impedance of the index finger when only one finger is used, as shown in Figure 6b, it is not applicable for using the conventional modeling technique. In addition, since the contact area of the index finger is vulnerable to motion artifact due to its connection to the TX and RX simultaneously, minimizing the measurement time is crucial. Figure 7 shows the device and electrodes used for experiments. As shown in Figure 7a,b, the TX and RX electrodes are arranged adjacent to each other to facilitate contact together. An example of actual measurement by touching the index finger is given in Figure 7c.

### 3.2. Data Acquisition Process

Table 1 shows the equipment used in this study. Two commercial products predicting PBF using BIA were used as a comparison group. Inbody-720 (Inbody Co. Ltd., Seoul, Korea) has a high correlation of 94% with DEXA, and the whole body measurement method is applied. In this study, this device was used as a reference device. Next, OMRON HBF-306 (Omron, Kyoto, Japan) using the upper half of the body was further used. Finally, 8 × 8 electrodes and hardware were used for this study. In this research, input data were composed of consecutively measured impedance data and anthropometric-based data. The measurement process was as follows. When the subjects arrived in the measurement room, the purpose of the experiment was explained, and their consent and demographic data were obtained using a simple questionnaire, which also sought to verify that the subjects were good candidates for having their measurements taken. First, anthropometric-based information, including height, weight, gender, and age, were measured. Waist and hip circumferences were used as additional features to reflect the characteristics of lower-body composition. Second, PBF was measured using commercial devices: Inbody-720 that measured the composition of the entire body, and OMRON HBF-306 that only measured the composition of the upper body. The measurements were repeated three times, and the mean values were used. Finally, upper-body impedance was measured using the proposed posture and the 8×8 mm^2^ electrodes depicted in Figure 7a,b, where we used a 50 kHz input current source, and impedance values were measured five times at one-second intervals. There was a 15-min break between each BIA measurement.

### 3.3. Subject Statistics

To validate the system performance in various types of human body, we collected body composition data (i.e., BIAs from 163 subjects, 102 males and 61 females). Ethics approval was received from the Institutional Review Board of Yonsei University; all participants provided written informed consent. Table 2 and Table 3 show the data from the subjects, where 141 subjects were athletes who played university sports, and 22 were non-athletes who exercised periodically, four or more times per week. Because PBF was measured using upper-body impedance, it was essential to analyze the variation in accuracy caused by different body shape. To include various body shapes, the study sample included both subjects whose preferred sports required lower-body muscle development (e.g., ice hockey, baseball, rugby, and taekwondo) and those that required upper-body development (e.g., kendo, table tennis, and judo). To increase the accuracy of the measurements, the subjects were prohibited from drinking alcohol and exercising vigorously for one day before the measurements. In addition, for one hour before the measurements, participants were prohibited from drinking any liquid or from doing any activity that would make them sweat.

### 3.4. Comparison of the PBF Results of Whole-Body and Upper-Body Measurements

**Figure 8:**
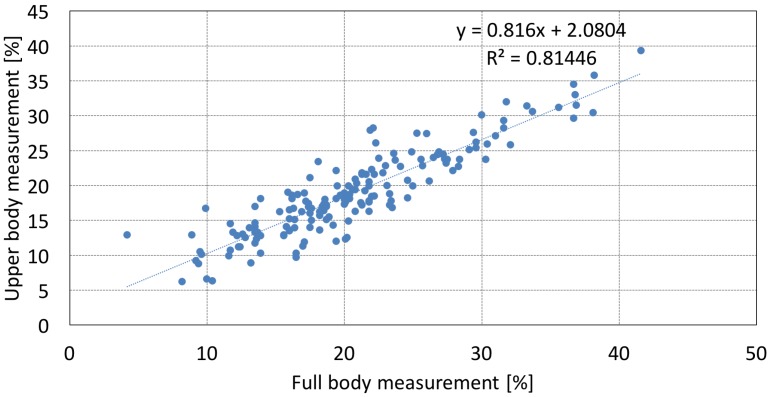
PBF measurement results using whole-body and upper-body measurements. The x-axis shows the results of whole-body measurements using Inbody-720. The y-axis shows the results of upper-body measurements using OMRON HBF-306.

The last lines of Table 2 and Table 3 describe the correlation values obtained by whole-body measurement with Inbody-720 and upper-body measurement with HBF-306, presented separately by the type of sport. Although the results were very good for most cases, the correlations were relatively low for basketball, kendo, ice hockey, and baseball players. The reason for the results could be the fact that the additional features might be irrelevant for subjects whose muscle or body shape differed from those of other subjects. Note that the height of the basketball players was 20 or more centimeters more than that of the other subjects, and the waist and hip circumferences of ice hockey and baseball players were very large. In other words, in cases where the lower body was overly developed or the subjects were disproportionate, it was difficult to determine whole-body PBF using only upper-body impedance values. In case of kendo, it showed the lowest BF among the experimental group. This means that if the BF is low, the error will also increase with the measurement method. Figure 8 plots the measurement results of both methods to illuminate the differences in performance between them. The measured values produced by the two methods differ, and these differences were caused by the upper-body measurement device not including any lower-body data.

## 4. Input Feature Processing Methods

Input feature processing methods consist of the input feature–calibration module and the PBF estimation module. The methods were designed to provide reliability to the measured data, to improve data accuracy, and to minimize the measurement time. The input feature–calibration module consists of three substeps: The validity of the measurement, the prediction of the settled value, and the generation of estimated features. The PBF estimation module is implemented with a deep neural network (DNN) framework. The details of the feature processing methods are described in the following subsection.

### 4.1. Signal Feasibility Checker

In this module, unreliable data caused by the user’s movement or variations in the surrounding environment are disregarded. Specifically, subjects receive a re-measurement flag if the two conditions as follows below are met.

Condition1: The measurement value continuously increases in one second time step. This condition prevents a situation where the measured values are not converged.

Condition2: The final measured value is outside the range of 500–2000 Ω. The range was set by experimental results.

### 4.2. Settling Value Estimator

**Figure 9:**
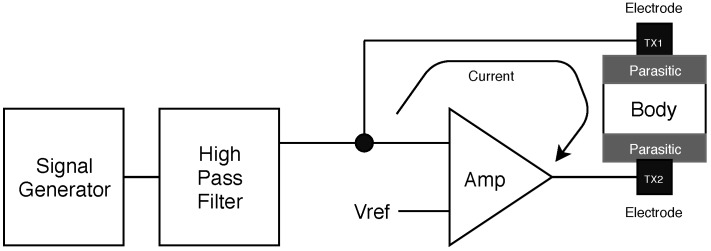
TX system diagram. The feedback loop is formed around the body and the circuit. Therefore, the parasitic component is an important component to determine the characteristics of the feedback loop.

The principle of the TX feedback loop is shown in Figure 9. The TX circuit generates a sine signal, passes the high-pass filter, and transmits an AC voltage of 50 kHz to the circuit. This voltage flows through the circuit configuration of the amplifier shown in Figure 9 when the two electrodes come into contact with the human body. At this time, the characteristics of the feedback loop are affected by the parasitic component in contact with the electrode. It takes a very long time to obtain a stabilized impedance value when a small electrode-based device is used because of the long convergence time caused by the small contact area on the skin. Furthermore, the convergence time varies by the contact state of the electrode and the dryness of the skin [40]. The convergence time is not a crucial issue in commercial systems, because the contact area of the electrodes on the skin is very large. As shown in Figure 2, the impedance measurement formed a long-range feedback loop while passing through the measuring circuit, electrode, skin, and human body. Ideally, the feedback loop should be affected only by the body impedance. However, in a real system, the settling time is further increased by various parasitic RC components, such as skin-to-electrode impedance. This feedback loop slowed down the convergence time, which was closely related to the value of parasitic RC. The following first-order step response represents the relationship:

(1) $$\begin{array}{c} v\left(t\right)=v_{0}\left(1-e^{-t/RC}\right)u\left(t\right). \end{array}$$

**Figure 10:**
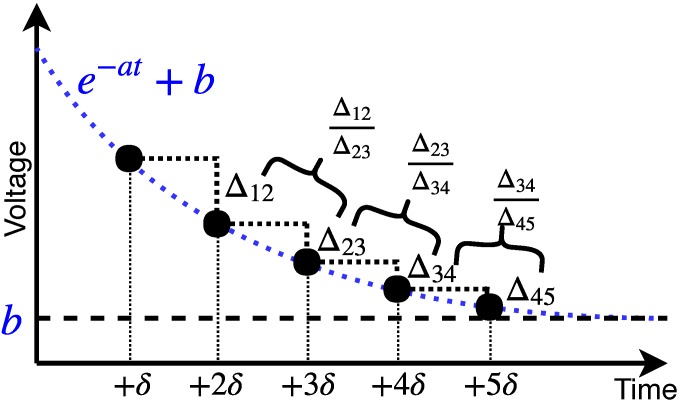
A method to estimate a settling value using initial measurement data (e.g., $\Delta_{12}$ = $V_{\delta }-V_{2\delta }$).

The RC is a parasitic value related to the contact area between the electrode and the body. The use of small electrodes in wearable devices results in a rapid increase of the resistor, R, which increases the overall time delay. In addition, a stable output cannot be obtained, as the value of R changes greatly with the user’s movement. In the present study, we set the target measurement time to be faster than five seconds. The threshold for determining the stabilized impedance was set to five ohms, which is the difference in value between four and five seconds. We introduced a method to predict the final convergence value using a large amount of initial measurement data, as shown in Figure 10.

The delay phenomenon in Equation (1) can be simplified to Equation (2):

(2) $$y\left(t\right)=e^{-at}+b,$$

where *a* is a constant value by RC and *b* is an output value to be converged. Generally, a problem arose that the value of *b* could not be predicted only with the measured value. To address this problem, a delta parameter was used. First, the difference between the value at *t* and t+1 was calculated; then, the delta values were calculated to eliminate the influence of the value of *b*, as follows:

(3) $$\Delta_{t,t+\delta }=\left(e^{-a(t+\delta )}+b\right)-\left(e^{-at}+b\right)=e^{-a(t+\delta )}-e^{-at}.$$

The ratio of variation of the delta value, $R_{\delta }$, was defined as follows:

(4) $$R_{\delta }=\frac{\Delta_{t,t+\delta }}{\Delta_{t+\delta ,t+2\delta }}=\frac{e^{a}t}{e^{-a(t+\delta )}}=\frac{1}{e^{-a\delta }}.$$

By setting the target value of $R_{\delta }$ close to 1, we could estimate the converged impedance value when the inverse process was performed through a log-based regression. In this study, five input values were measured at one-second intervals, and the threshold value of the $R_{\delta }$ was set at 0.95–1.05.

### 4.3. Predicting the Value of $H^{2}/R_{50}$

The proposed method is not applicable for predicting the cylinder-based impedance value discussed in Section 2.2 because the measurement also contains the large impedance value caused by a finger. To address this problem, we predicted the output impedance value by utilizing $H^{2}/R_{50}$ through a process of estimating the impedance-settling value. We introduced a neural network-based approach because its performance is superior to the conventional linear regression algorithms when the number of input features is large and they do not have linear relationships. The input features are impedance values, height, age, gender, weight, waist circumference, and hip circumference. Three hidden layers consisting of 128 nodes were used, as shown in Table 4. The final output parameter was defined as the value of $\hat{H^{2}/R_{50}}$.

### 4.4. PBF Estimation Method Using Deep Neural-Networks

The ultimate goal of the present study was to estimate PBF values using only upper-body impedance. To reflect the characteristics of the lower body’s composition, we included waist and hip circumferences as auxiliary features.

To evaluate the impact of each input feature, including the proposed waist and hip circumference to the estimated output parameter, we implemented a linear regression model conventionally used in this type of study [42]. Table 5 summarizes the estimated features and coefficients. In general, the *p*-value is used to determine the significance of applied hypotheses. All the conventional features have low *p*-values (<0.05), meaning that they are highly correlated [43]. However, the *p*-value of the newly added waist and hip information is higher than 0.05, which confirms that it is challenging to model anthropometric-based data using the linear regression method.

To address this limitation, we proposed a DNN-based technique to reliably estimate PBF. The proposed system consists of three hidden layers, and each hidden layer has 256 nodes. A rectified linear unit (ReLU) was used as an activation function. We used an early-stopping method to minimize the overfitting effect because the number of data that we could utilize was not sufficient. The results were not much different when we introduced a dropout method. The DNN-based algorithm successfully predicted the body composition of whole-body only with the measured impedance of upper-body only. Recall that the waist and hip circumferences were added to the network to supplement the data on lower-body impedance.

## 5. Results and Discussion

### 5.1. Input Feature Calibration Results

**Figure 11:**
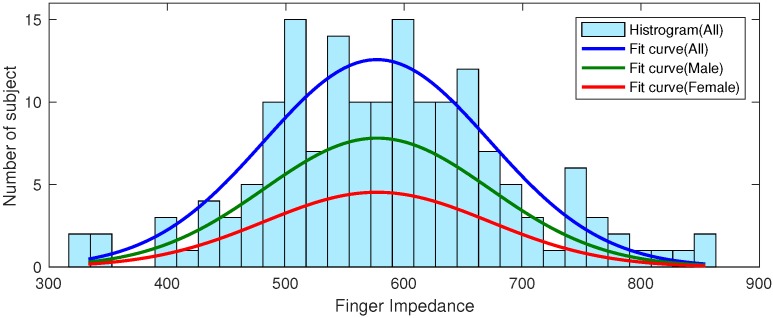
Finger impedance measurement data. The settling algorithm was applied to the measured values, and the impedance was calculated by subtracting the upper-body impedance measured using a large-sized electrode. There was no gender difference.

To evaluate the performance of the proposed calibration method, we calculated the correlation coefficient between the reference impedance value obtained with large-sized electrodes and the one obtained with the proposed small-sized electrodes. The correlation coefficient was 0.74 when no calibration was made with the five-second measurement time, but it went up to 0.77 when the proposed calibration was applied. Experimental results showed that when the calibration was made, the corrected value became 35 ± 43 Ω, which indicated the effectiveness of the calibration process. Although the improvement in terms of correlation coefficient was minor, it was still important to improve the performance of the following DNN modeling process. Note that it was very difficult to obtain a high correlation value because it would inevitably contain undesirable impedance caused by fingers. Note that the impedance value of the index finger is larger than that of the upper half of the body. Figure 11 shows that the measured finger impedance was 585.06 ± 102.67 Ω for all subjects: 576.96 ± 95.36 Ω for males and 598.88 ± 113.54 Ω for females. No gender-specific differences were observed. Therefore, it was still unsuitable for estimating the $H^{2}/R_{50}$ value with the conventional cylinder-based model framework. Consequently, we included height, age, gender, and weight as additional input features and adopted a DNN model to correctly estimate the $H^{2}/R_{50}$ value.

Table 6 shows multiple experimental setups. Table 7 shows the correlation coefficients calculated by reference and estimated values. The conventional system used the impedance value of five seconds. When the calibrated impedance value used the algorithm described in Section 4.2, the correlation coefficient improved by 37.2%. As shown in the columns for Models I–III, all the DNN techniques showed better results than the linear regression model. Finally, we obtained the best performance when the waist and hip circumferences were added to the DNN input features. These results confirmed that the additional features related to lower-body composition were helpful for estimating whole-body impedance.

### 5.2. PBF Estimation Using Deep Neural Networks

Among 163 subjects that we collected for processing, 143 were used for training and 20 (10 males and 10 female) for test data. The data set was chosen to have a balanced distribution. The $r^{2}$ and SEE values were used to evaluate performance because they sufficiently represent linearity and distribution [29,30,31,32,33,34,35,36]. The DNN-based method was used to estimate $H^{2}/R_{50}$, and Model III, which used waist and hip circumferences, was also adapted to compensate for bias caused by using upper-body impedance only.

**Figure 12:**
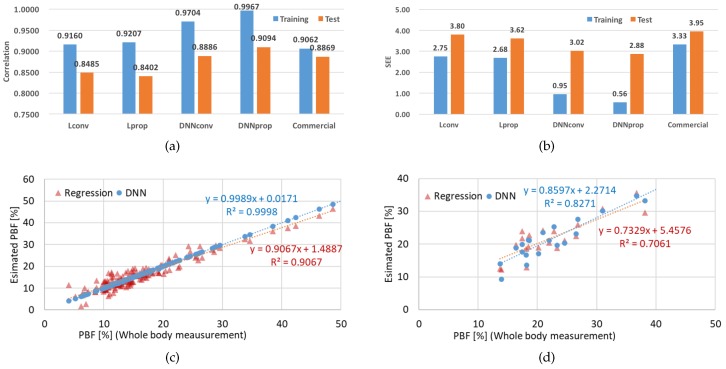
PBF estimation results for models based on regression and DNN. (**a**) Correlation coefficients obtained from each method. For the label information, “L” means linear regression and “DNN” means deep neural network. The difference due to the input feature is distinguished by the “conv” and the “prop”. The “conv” is composed of the parameters used in the previous research, and “prop” is applied around the waist circumference and the hip circumference; (**b**) standard error of estimate (SEE) values obtained from each method; (**c**) training results obtained from 143 subjects; (**d**) test results obtained from 20 unseen subjects. Commercial device data were taken from HBF-306.

Table 8 summarizes the comparison results. First, we analyzed the results of adding waist circumference and hip circumference to reduce the limitations of using only upper-body impedance. L_conv_ is a method based on features from previous studies using linear regression. L_prop_ added waist circumference, hip circumference, and waist to hip ratio to the L_conv_ technique. Figure 12 shows the comparison results between the reference and estimated PBF ratios for the training and test sets. The results showed that the L_conv_ and L_prop_ are similar in terms of performance, as shown in Figure 12a,b. As mentioned in Section 4.4, it was confirmed that the linear regression modeling technique does not represent the waist circumference and the hip circumference, which are assumed to present lower-body information. Yet, when DNN was applied, both training and test results improved. The results of the training set and the test set are shown in Figure 12c,d. The x-axis is the ground truth and the y-axis is the estimated output. From the training results shown in Figure 12c, it can be seen that the DNN technique has a higher correlation than the regression model. This confirms that DNN is more efficient when the anthropometric data proposed in this study are included. Further, the results of the test set which were not used in the process of network training are shown in Figure 12c. In this experiment, it was confirmed that the DNN technique shows a higher correlation value than the conventional regression technique.

The results of the proposed PBF prediction system were $r^{2}$ = 0.9094 and SEE = 2.88. It was confirmed that the correlation coefficient was higher than that of the value from the commercial device, which measured only upper-body impedance. This BF prediction system based on DNN showed 9% higher accuracy for $r^{2}$ values and a 28% reduction in SEE compared to the conventional linear-regression model. It is clear that the DNN technique better represents the influence of various features. However, we still think that the amount of database needs to be increased and a more effective network structure should be designed to further increase accuracy.

## 6. Conclusions

We proposed a system that predicts PBF quickly and accurately using upper-body impedance values and anthropometric data. To enhance the reliability of the results, experiments were performed using 163 subjects whose body shapes were classified into several types. The proposed device was comfortable because measurements were taken by contacting a single finger only. To decrease the measurement time while maintaining its accuracy, the proposed system used only five impedance values and measured one value at every second with an effective calibration method. The test results validated that the inclusion of anthropometric data was helpful for improving accuracy, especially when a deep learning approach was used to predict the regression values.

It is possible to expand the proposed structure to IoT-based sensor systems. Using a small-sized DNN network, the sensing device would transmit optimized features to a server. The server would then configure a large-sized DNN network to guarantee good feedback performance. This configuration enables the deployment of various types of application systems more efficiently and accurately.

## Figures and Tables

**Table 1:** Comparison of measurement device.

Items	Unit	Inbody-720 (Reference Device)	HBF-306	This Work
Measurement Point		Whole body	Upper body	Upper body
TX Frequency	kHz	1, 5, 50, 250, 500, 1000	50	50
Electrode	EA	8	4	4
Output Information		ICW ECW Dry Lean Mass Body Fat Mass	Body Fat Ratio	Body Fat Ratio
Accuracy (PBF)	%	94% with DEXA [39]		

**Table 2:** Statistical analysis data for males.

	Summary	Basketball	Kendo	Ice Hockey	Baseball	Rugby	General
	102	9	13	10	22	32	16
Height (cm)	178.0 ± 7.2	190.0 ± 7.2	172.0 ± 7.7	176.9 ± 3.7	176.4 ± 4.7	179.6 ± 6.4	174.5 ± 5.7
Age (year)	21.3 ± 3.1	20.3 ± 1.6	19.1 ± 0.6	20.1 ± 1.1	21.0 ± 3.1	20.1 ± 1.4	26.2 ± 3.0
Weight (kg)	83.6 ± 13.0	84.6 ± 10.6	70.5 ± 8.3	81.7 ± 4.8	79.4 ± 10.4	93.1 ± 15.0	78.7 ± 9.8
Waist (cm)	83.5 ± 7.8	79.0 ± 3.6	78.5 ± 5.2	83.1 ± 4.0	82.2 ± 7.7	86.2 ± 14.0	81.4 ± 6.7
Hip (cm)	100.7 ± 6.4	97.8 ± 8.6	94.6 ± 4.5	102.8 ± 1.8	99.4 ± 5.2	104.4 ± 6.8	99.2 ± 5.0
BF (%)	17.9 ± 5.9	10.0 ± 2.7	15.3 ± 3.7	18.1 ± 2.9	17.3 ± 4.2	21.6 ± 6.8	18.3 ± 5.1
BMI	26.3 ± 3.5	23.3 ± 1.9	23.8 ± 1.8	26.2 ± 1.7	25.5 ± 3.1	28.8 ± 4.2	25.8 ± 2.6
Correlation*	0.8385	**0.0489**	**0.4925**	**0.6574**	0.7690	0.8797	0.9491

Correlation*: Correlation coefficient between whole-body measurement using Inbody-720 and upper-body measurement using HBF-306.

**Table 3:** Statistical analysis data for females.

	Summary	Basketball	Kendo	Taekwondo	Judo	Table Tennis	General
	61	8	4	14	23	6	6
Height (cm)	165.2 ± 6.2	167.0 ± 4.6	166.0 ± 3.0	167.3 ± 5.1	163.8 ± 8.0	161.6 ± 3.5	166.9 ± 4.2
Age (year)	20.2 ± 2.4	20.9 ± 1.7	20.0 ± 1.4	19.1 ± 1.2	20.0 ± 1.4	20.2 ± 1.3	24.6 ± 6.1
Weight (kg)	65.4 ± 13.5	66.2 ± 9.5	62.6 ± 3.1	61.1 ± 9.4	72.0 ± 17.7	55.8 ± 6.0	62.4 ± 4.6
Waist (cm)	74.2 ± 8.8	73.3 ± 6.5	71.8 ± 3.5	70.4 ± 6.9	77.9 ± 10.0	73.6 ± 5.9	73.7 ± 13.2
Hip (cm)	97.7 ± 6.5	100.0 ± 5.9	95.0 ± 2.5	96.8 ± 5.4	99.8 ± 7.9	92.9 ± 5.0	95.4 ± 3.3
BF (%)	25.2 ± 6.0	26.8 ± 7.6	21.4 ± 4.2	23.2 ± 5.4	26.8 ± 6.7	25.8 ± 4.8	23.5 ± 4.5
BMI	24.1 ± 4.3	24.9 ± 5.2	22.7 ± 1.7	21.8 ± 3.3	26.6 ± 4.6	21.4 ± 2.3	23.0 ± 1.6
Correlation*	0.9201	0.9479	0.9909	0.9086	0.9337	0.9344	0.7773

Correlation*: Correlation coefficient between whole-body measurement using Inbody-720 and upper-body measurement using HBF-306.

**Table 4:** Information of neural networks for estimation of $H^{2}/R_{50}$ and percent body fat.

	Network 1	Network 2
	($H^{2}/R_{50}$)	(PBF)
Training Set	143 (male: 93, female: 50)	
Test Set	20 (male: 10, female: 10)	
Input layer	7	8
Hidden layer	3	3
Output layer	1	1
Hidden node	128 × 128 × 128	256 × 256 × 256
Activation function	ReLU	
Optimizer	Adam Optimizer [41] (beta1 = 0.9, beta2 = 0.999, epsilon = 1 × 10^−8^)	
Cost function	Mean square error (MSE)	
Number of training cycles	maximum = 2000 (Early stopping adopted)	

**Table 5:** Comparison of linear regression model between conventional and proposed features.

	Conventional Features		Proposed Features	
	Coefficient	*p*-Value	Coefficient	*p*-Value
Intercept	59.6240	<0.0001	−16.2929	**0.6774**
Age	−0.0992	**0.2170**	−0.1302	**0.1059**
Gender	7.4604	<0.0001	7.5189	<0.0001
Height	−0.4174	<0.0001	−0.4005	<0.0001
Weight	0.4673	<0.0001	0.3763	<0.0001
$H^{2}/R_{50}$	−0.4217	<0.0001	−0.4021	<0.0001
Waist	–	–	−0.6804	**0.1224**
Hip	–	–	0.7177	**0.0674**
Waist to Hip	–	–	78.5731	**0.0837**

**Table 6:** $H^{2}/R_{50}$ Estimation parameter.

Case	Methods	Input Dimension	Detailed Information
Conventional	Regression	5	H, A, G, W, $R_{50}$_5
Estimated Impedance	Regression	5	H, A, G, W, $R_{50}$_prop
Model I	DNN (Network1)	5	H, A, G, W, $R_{50}$_prop
Model II	DNN (Network1)	6	H, A, G, W, $R_{50}$_prop, H${}^{2}$/Imp
Model III	DNN (Network1)	7	H, A, G, W, $R_{50}$_prop, H${}^{2}$/Imp, W/H

H: Height, A: Age, G: Gender, W: Weight, $R_{50}$_5: Impedance value at 5 s. $R_{50}$_prop: Impedance value adopting proposed algorithm, H^2^/Imp: $Height^{2}/R_{50}\_prop$, W/H: waist to hip ratio.

**Table 7:** $H^{2}/R_{50}$ Estimation results related with Table 5.

	Conventional	Estimated Imp.	Model I	Model II	Model III
Correlation*	0.8420	0.9008	0.9344	0.9338	0.9403
Improvement [%]	-	37.2	58.4	58.1	62.2

Correlation*: Correlation coefficient between the Inbody-720 device and proposed methods.

**Table 8:** Percent body fat estimation parameter.

Initial	Methods	Input Dim.	Detailed Information
L_conv_	Regression	5	H, A, G, W, H^2^/$R_{50}$
L_prop_	Regression	8	H, A, G, W, H^2^/$R_{50}$, hip Circ., waist Circ., waist/hip
DNN_conv_	DNN (Network2)	5	H, A, G, W, $\hat{H^{2}/R_{50}}$
DNN_prop_	DNN (Network2)	8	H, A, G, W, $\hat{H^{2}/R_{50}}$, hip Circ., waist Circ., waist/hip

H: Height, A: Age, G: Gender, W: Weight. $R_{50}$: Impedance value adopt proposed algorithm, Circ.: Circumference.

## Abbreviations

The following abbreviations are used in this manuscript:

BIA	Body impedance analysis
PBF	Percent body fat
SEE	Standard error of estimate
DEXA	Dual-energy X-ray absorptiometry
BMI	Body mass index
TBW	Total body water
FFM	Fat-free mass
FM	Fat mass
ReLU	Rectified linear unit
